# Variation in HIV-1 Tat and Vpr protein amino acid sequences and its association with vascular health measures in a South African cohort: an exploratory study

**DOI:** 10.1186/s12985-025-02891-8

**Published:** 2025-08-04

**Authors:** Esmé Jansen van Vuren, Yolandi Breet, Adriaan Jacobs, Iolanthé M. Kruger, Monray Edward Williams

**Affiliations:** 1 Hypertension in Africa Research Team (HART), North-West University, Potchefstroom, South Africa; 2 South African Medical Research Council: Unit for Hypertension and Cardiovascular Disease, North-West University, Potchefstroom, South Africa; 3 Africa Unit for Transdisciplinary Health Research (AUTHeR), North-West University, Potchefstroom, South Africa; 4 Biomedical and Molecular Metabolism Research (BioMMet), North-West University, Potchefstroom, South Africa

**Keywords:** Cardiovascular disease, Central haemodynamics, Arterial stiffness, HIV, Viral proteins

## Abstract

**Objectives:**

Human immunodeficiency virus (HIV)-1 is associated with adverse cardiovascular-related outcomes. Subtype-specific variations in the amino acid sequences of Tat and Vpr HIV-1 proteins are associated with differential clinical outcomes in people living with HIV (PLHIV). Given the diverse clinical outcomes related to different HIV subtypes, it is crucial to evaluate the variations in the sequence of Tat and Vpr amino acids in geographical regions where subtype C predominates, such as South Africa. This study aimed to determine whether specific Tat and Vpr protein amino acid variants (alone or in combination) are associated with vascular health measures and predict incident hypertension and all-cause mortality over a five-year period.

**Methods:**

A cohort of *n* = 60 treatment-naïve PLHIV at baseline and *n* = 35 at a five-year follow-up was investigated. Standardized vascular health measures, including carotid intima-media thickness (cIMT), cross-sectional wall area (CSWA) and carotid-radial pulse wave velocity (crPWV), as well as Sanger sequencing for Tat/Vpr analysis, were performed. The associations of vascular health measures with Tat and Vpr amino acid variants were investigated.

**Results:**

We found that the variation in amino acid sequence in Tat only (*p* = 0.039) and Tat/Vpr (*p* < 0.001) were associated with crPWV at baseline. Variation in the Tat and Vpr amino acid sequence did not predict incident hypertension in five years or all-cause mortality.

**Conclusion:**

The variants of the Tat and Vpr amino acid sequence were associated with arterial stiffness, which may be an underlying mechanism for cardiovascular disease development in PLHIV.

**Supplementary Information:**

The online version contains supplementary material available at 10.1186/s12985-025-02891-8.

## Introduction

Human immunodeficiency virus (HIV)-1 remains a global health concern, with nearly 39 million people infected according to the 2023 UNAIDS report [1]. Although HIV-1 is well known for the development of acquired immune deficiency syndrome (AIDS), it is also responsible for a wide range of clinical disorders, including, but not limited to, HIV-associated neurocognitive disorders (HAND) [2], HIV-related cancers [3] and HIV-associated cardiovascular disease (CVD) [4, 5].

The role of HIV-1 in the development of CVD is well-established [5], but the specific impact of HIV-1 subtype variation, particularly in the amino acid sequences of viral proteins, has been less explored. Research on HIV-1 protein variation in South Africa, where subtype C predominates, remains limited [6], with most studies focusing on geographic regions where other HIV subtypes predominate. Two proteins are of particular interest in this study, namely the regulatory protein, transactivator of transcription (Tat), and the accessory protein, viral protein R (Vpr) due to their known involvement in the cardiovascular system [7, 8].

The Tat protein is 86–101 amino acids in length and enhances viral transcription by binding a structured RNA element (TAR, transactivation-responsive region) present at the 5′-end of viral leader mRNAs [9, 10]. The Tat protein is released from Tat-expressing cells into the extracellular environment and modulates lymphocyte functions, promotes cell migration, and exerts cytopathic effects on leukocytes and neural cells [11, 12]. In the cardiovascular system, Tat is actively secreted into vascular endothelial cells, possibly contributing to endothelial dysfunction, excessive inflammation, and atherosclerosis [13]. This is supported by fundamental studies that have demonstrated the impact of HIV Tat on the cardiovascular system [14, 15].

Vpr plays a multifunctional role in the pathogenesis of HIV by aiding the nuclear localization of pre-integration complex [16], inducing cell cycle arrest of infected cells at G2/M phase [17], activating viral and heterologous gene transcription [18, 19] and promoting neuroinflammation [18, 19]. Furthermore, in clinical studies, Vpr has been shown to influence disease progression [20–22] and neurocognitive performance in people living with HIV (PLHIV) [23]. While research on the role of Vpr in the cardiovascular system is limited, studies in mice link it to atrial cardiomyocyte mitosis, mesenchymal tumours, dysrhythmia, heart failure [8] and heart tissue senescence [24].

Tat and Vpr are also known to interact functionally, modulating viral genome transcription [25] and host cell apoptosis [26]. Therefore, it is plausible that Tat and Vpr may function in a synergistic way to contribute to HIV-1-related clinical manifestations like CVD. Furthermore, specific amino acids within the Tat and Vpr proteins enhance viral activity [10, 22] potentially leading to increased viral loads and affecting mortality rates in PLHIV.

This study aimed to investigate whether specific variants of Tat and Vpr protein amino acids (in combination or alone) associate with vascular health measures and predict incident hypertension at five years as well as all-cause mortality in a cohort of South African PLHIV.

## Materials and methods

### Cohort characteristics

**Fig. 1:**
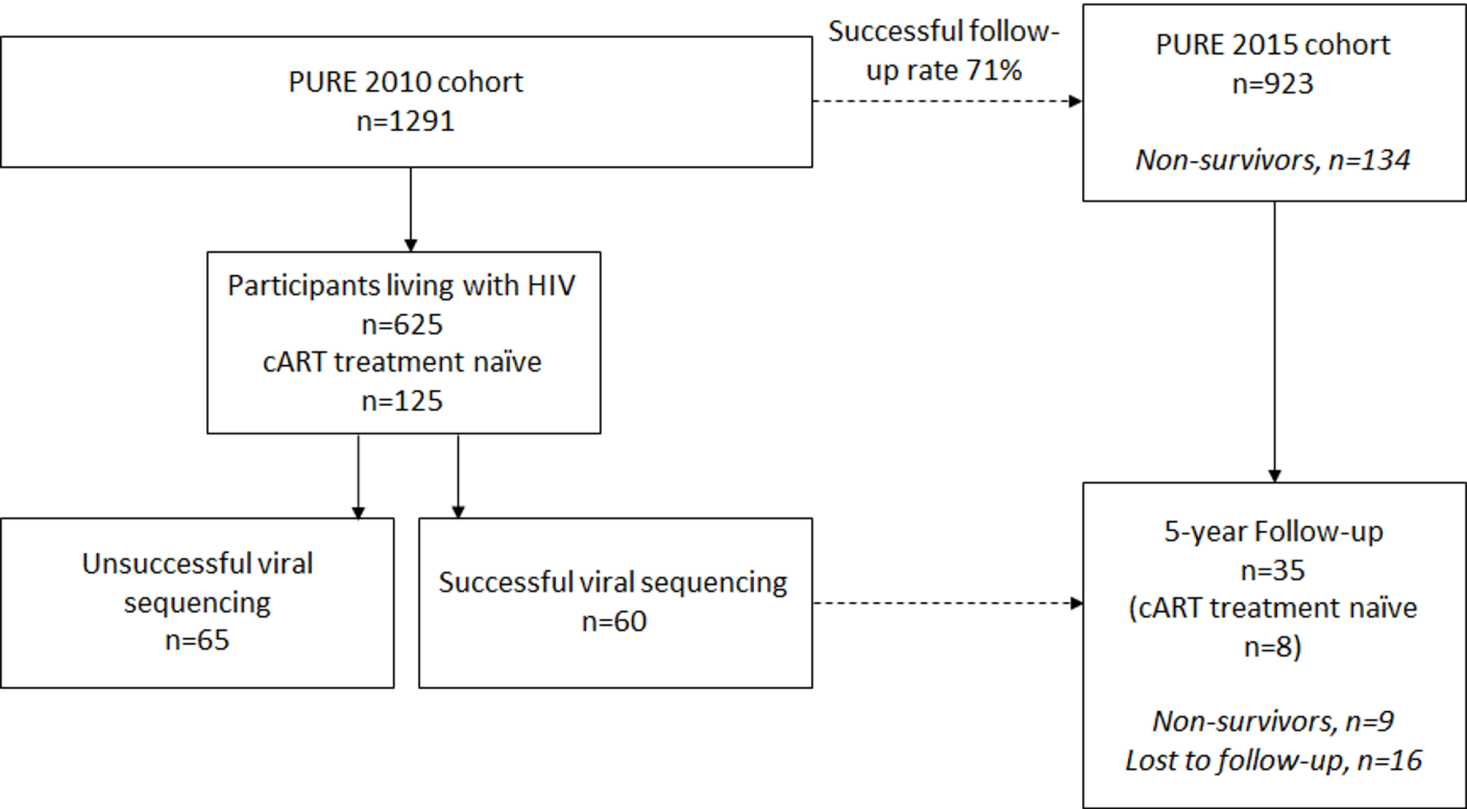
Study cohort design and participant follow-up flow diagram

The multinational Prospective Urban and Rural Epidemiology (PURE) Study is a prospective investigation designed to evaluate the development of CVD in individuals from 17 countries, representing high-, middle-, and low-income regions [27]. This study included men and women (aged > 30 years) of African descent that resided in two urban and two rural areas in the North-West Province of South Africa. After obtaining voluntary informed consent, baseline data collection took place in 2005 (*n* = 2010) with follow-up data collected in 2010 (*n* = 1291) and 2015 (*n* = 923). For this study, we selected participants from the 2010 leg of the study (*n* = 125) who were living with HIV but were not treated with combination antiretroviral therapy (cART). We analysed data from 2010 to 2015 for these participants. Therefore, this study presents a unique treatment naïve cohort at baseline (in 2010), as we wanted to sequence the HIV-1 viral proteins without the confounding influence of antiretroviral therapy (cART in 2015 was accounted for in statistical analyses). Sequencing of viral genes (proteins) was successful for 60 participants (50%), as shown in Fig. 1. Baseline demographic and cardiometabolic characteristics of participants with successful sequencing were compared to those without, as detailed in Supplementary Table 1. HIV-1 in all participants was classified as subtype C after subtyping using COMET [28]. Tat sequence data were available for *n* = 37 participants and Vpr sequence data were available for *n* = 47 participants. Both the protocols for the South African leg of the PURE Study (NWU-00016-10-A1) and this sub-study (NWU-00106-22-A1) was approved by the North-West University Health Research Ethics Committee (NWU-HREC) in South Africa.

### HIV status

Participants received group counselling by trained counsellors prior to their HIV status being determined. The HIV status was determined following the South African Department of Health protocol, utilizing the First Response rapid HIV card test (Premier Medical Corporation Limited, Daman, India). In the event of a positive result, the test was confirmed with the SD BIOLINE HIV 1/2 3.0 card test (Standard Diagnostics, INC, Korea). Finger prick blood and a point-of-care device (PIMATM CD4, Alere, Jena, Germany) with fixed volume cytometry analysis were used to determine the CD4^+^ cell (helper T-cell) count. Regardless of their results, each participant received individual post-counselling and, if a positive confirmation was obtained, a follow-up and CD4 + cell count analysis was done by referring the participant to the nearest clinic or hospital. Treatment was then initiated at the clinics as part of a roll-out program, with the regimen consisting of two nucleoside reverse transcriptase inhibitors (Stavudine and Lamivudine) and one non-nucleoside reverse transcriptase inhibitor (Efavirenz or Nevirapine) [29].

### General demographics and anthropometric measurements

The PURE South Africa Adult Questionnaire was used to obtain basic demographic (age and sex assigned at birth) and lifestyle information (self-reported tobacco and alcohol use) as well as medical history of the participants and use of cART and antihypertensive medications. Anthropometric measurements were performed in accordance with the guidelines of the International Society for the Advancement of Kinantropometry and included body height (Invicta Stadiometer, IP 1465, Invicta, London, UK), body weight (Electronic scale, Precision Health Scale, A&D Company, Tokyo, Japan) and waist circumference (Holtain unstretchable metal tape). Body mass index (BMI) was calculated as weight (kg) / height (m)^2^.

### Vascular health measures

Participants were asked to refrain from smoking, exercising or eating for at least 30 min prior to cardiovascular assessments. Office brachial blood pressure measurements were obtained with Omron devices (Omron Healthcare, Kyoto, Japan) in 2010 (Omron HEM-757) and 2015 (OMRON M6) while participants were in a seated position with their right arm supported at the heart level. After a ten-minute rest period, measurements were taken twice on the participant’s right upper arm, over the brachial artery, using an appropriately sized cuff. There was a five-minute rest period between each measurement. The second measurements of the brachial systolic blood pressure (bSBP), diastolic blood pressure (bDBP) and heart rate were used for statistical analyses. The brachial pulse pressure (bPP) was calculated as the difference between bSBP and bDBP, while the brachial mean arterial pressure (bMAP) was calculated with the following formula: bMAP=(bSBP(2xbDBP))/3. Hypertension was defined according to the 2013 ESH/ESC guidelines as bSBP ≥ 140 mmHg, bDBP ≥ 90 mmHg and/or use of antihypertensive medications. Central systolic blood pressure (cSBP) and pulse pressure (cPP) were measured with the Omron 9000AI device (Omron Healthcare, Kyoto, Japan) device in 2010 and the SphygmoCor XCEL System (AtCor Medical Pty Ltd., Sydney, Australia) in 2015. Carotid-radialis pulse wave velocity (crPWV), as a measure of arterial stiffness, was determined on each participant’s left side while in a supine position, with the Complior SP device (Artech-Medical, Pantin, France) in 2010. Since a different arterial segment and device were used for the measurement of arterial stiffness during the 2015 data collection (carotid-femoral PWV; SphygmoCor^®^ XCEL System, AtCor Medical Pty. Ltd, Sydney, Australia) changes over time for PWV, as well as cSBP and cPP, could not be calculated in this study. The near and far wall measurements of carotid intima-media thickness (cIMT) were recorded in duplicate in 2010 and 2015 using the SonoSite Micromaxx ultrasound system, SonoSite, Inc., Bothel, WA, USA, and the average of all the measures were used. Digitalised images were analysed in 2010 and 2015 with the Artery Measurements Systems software (I version 1.139, Chalmers University of Technology, Gothenburg. Sweden BP: OMRON HEM-757 device (Omron Healthcare, Kyoto, Japan). The carotid cross-sectional wall area (CSWA) was calculated with the following equation: CSWA = [3.14x(d/2 + cIMTf)x(d/2 + cIMTf)]-[3.14x(d/2)x(d/2)], where d = luminal diameter.

### Mortality assessment

Trained fieldworkers contacted the participants and conducted regular home visits (every three months) over the five-year period after the first follow-up data collection was conducted in 2010. Verbal autopsy and death certificates were used to obtain the cause of death, and a physician coded the immediate and underlying causes according to the International Classification of Diseases (ICD-10) codes.

### Biochemical analyses

Samples had to be collected after a fasting period. They were prepared within 2 h of collection by: (1) centrifugation (2000 x g, 15 min, 10 °C) (2), transfer to microcentrifuge tubes and freezing using dry ice, and (3) storage at -80 °C for further analysis. The sample preparation for samples collected in rural areas was as follows: (1) samples were frozen immediately using dry ice (2), stored at -18 °C until transportation to the laboratory could be arranged (maximum of 5 days), and (3) once at the laboratory, stored at -80 °C for further analysis.

#### Metabolic and inflammatory biomarkers

The Cobas Integra 400 plus (Roche Diagnostics, Basel, Switzerland) was used to analyse total cholesterol (TC), high-density lipoprotein cholesterol (HDL-C), gamma-glutamyl transferase (GGT), and C-reactive protein (CRP) levels in serum samples, with intra- and inter-coefficients of variation for all assays being below 10%. Percentage glycated haemoglobin (HbA1c) was analysed in plasma with an ion-exchange high-performance liquid chromatography method, D-10 Haemoglobin testing system from Bio-Rad (#220 − 0101).

#### Viral protein analysis

Tat and Vpr amino acid sequences were determined using Sanger sequencing as described in our previous studies [30, 31]. RNA was extracted from plasma samples, reverse transcribed and the region Tat exon 1/Vpr (HXB2 position 4900–6351) was amplified. All DNA sequences were analysed using the ABI Prism 3130xl automated DNA sequencer (Applied Biosystems, Foster City, CA). The resulting sequences were translated to the corresponding amino acid sequences [30, 31]. Sequences are available in GenBank with the accession numbers OR621303-OR621349 for Vpr and OR645413-OR645449 for Tat.

We assigned composite risk scores for Tat and Vpr variants, similar to previous studies [23, 32]. Amino acids were classified based on their potential influence on cardiovascular risk, with higher scores indicating higher risk. Specifically, for Tat, high-risk amino acids (score = 2) were Lysine (K)24, Cysteine (C)31, Arginine (R)57, and Leucine (L)68, while low-risk amino acids (score = 0) were Asparagine (N)24, Serine (S)31, S57, and Proline (P)68. For Vpr, high-risk amino acids (score = 2) were Tyrosine (Y)45, Alanine (A)55, Isoleucine (I)63, R77, and R85, while low-risk amino acids (score = 0) were Histidine (H)45, Threonine (T)55, T63, Glutamine (Q)77, and Proline (P)85. Any other amino acids at the investigated positions were classified as neutral risk (score = 1). The selected amino acids have demonstrated influence on other pathophysiological pathways [22, 23, 33, 34] and may have overlapping roles in the development of CVD, warranting further analysis. A composite risk score was generated by summing the assigned values for each protein.

### Statistical analysis

All analyses were performed using IBM SPSS (version 28, IBM, USA). *P*-values < 0.05 were considered statistically significant for all analyses. GraphPad Prism version 5.03 (GraphPad Software Inc., CA, USA) was used for the graphical illustration of the data. All variables were assessed for normality by the visual inspection of QQ-plots and using descriptive statistics. Non-parametric data were logarithmically transformed (BMI, CRP, GGT, and HDL-C). Categorical data are presented as frequencies and proportions while continuous data are reported as mean ± standard deviation if the variables follow a normal distribution, or the median with the interquartile range for log-transformed data.

Differences over time were calculated using dependent t-tests for continuous variables and McNemar’s test for categorical variables. Backward multiple regression analyses were used to explore cross-sectional (crPWV, cIMT, CSWA, bSBP, bDBP, bPP, bMAP, cSBP, cPP) and longitudinal (%∆cIMT, %∆CSWA, %∆bSBP, %∆bDBP, %∆bPP, %∆bMAP) independent associations of vascular health measures with Tat and Vpr sequence risk scores while adjusting for confounders. Pearson correlations and backward multiple regression analyses were performed to determine which confounders to be included in the statistical models. Variables that did not show a significant correlation with the dependent variables or did not enter the final models in the case of the regression analyses were excluded to minimise the risk of statistical model overfitting. Confounders that were considered included age, sex, locality (rural, urban), TC: HDL-C ratio, CRP, alcohol use (yes, no) or GGT (as an indicator of alcohol use), tobacco use (yes, no), BMI, CD4 + count, HbA1c, bMAP (in the case of crPWV and cIMT only), use of anti-hypertensive medication (yes, no) and duration of cART use or use of cART at follow-up (yes, no; for longitudinal analyses only). Percentage change (%∆) over the five-year period was calculated with the following formula: %∆ = (follow-up − baseline)/baseline*100. Logistic regression analyses were conducted independent of confounders to determine whether Tat and Vpr sequence scores increases the likelihood for hypertension at baseline (2010). The predictive value of baseline HIV-1 Tat and Vpr protein sequence variation (risk scores) for five-year incident hypertension and all-cause mortality risk was investigated using multivariable adjusted Cox regression analyses to compute standardised hazard ratios; with the time variable calculated for each participant as the number of days between the 2010 data collection date and the 2015 data collection date or date of death, respectively.

## Results

### Sample characteristics

This study included a total of *n* = 60 treatment naïve PLHIV at baseline with a median age of 46 years as shown in Table 1. A total of 28.7% (*n* = 16) of the cohort were men, 51.7% resided in a rural area (*n* = 31) and 41.9% (*n* = 26) had hypertension at baseline. No viral load data was recorded in the primary study; however, all participants were cART treatment-naïve at baseline sample collection. CD4 + count data were available for 59% of participants, with a mean CD4 + count of 302 cells/µl. The mean scores for Tat/Vpr, Tat only and Vpr only were 7.72 (± 1.95), 2.72 (± 1.39) and 5.06 (± 1.31) respectively.

Changes in the basic characteristics and vascular health measures over the five-year period are presented in Table 2. Participants revealed increases for GGT (*p* = 0.04), TC (*p* = 0.02) and HDL-C (*p* = 0.001) from baseline to follow-up. Significant decreases were observed in HbaA1c and cIMT (*p* = 0.017). Of the 26 individuals that had prevalent hypertension at baseline (Table 1), nine no longer had hypertension at follow-up, as shown in Table 2. Conversely, seven individuals who were normotensive at baseline developed hypertension during follow-up. This results in seven cases of incident hypertension and eight individuals with sustained hypertension over the five-year follow-up period. During the five-year period, 27/35 (77.1%) participants started to use cART medication, with 8/35 (22.9%) participants having never used cART medication (*p* < 0.001). Date of cART initiation was only available for 37% (13/35) of the participants. The proportion of participants that used tobacco and alcohol at baseline and follow-up also did not differ (*p* = 0.15 and *p* = 0.50, respectively) (data not shown). Over the five-year follow-up period, nine individuals passed away. The causes of death were primarily non-specified HIV-related conditions, along with meningitis, hepatitis, tuberculosis, and renal failure. Notably, none of the deaths were attributed to cardiovascular causes.

**Table 1:** Baseline characteristics of HIV-positive study participants

	Total cohort (*N* = 60)
Sex (male), n (%)	16 (26.7)
Locality (rural), n (%)	31 (51.7)
Hypertension (yes), n (%)	26 (41.9)
Use of diabetic medication (yes), n (%)	1 (1.7)
Age, mean *	46 (8)
CD4 + Nadir, cells/mm^3^	302 (± 149)
Tat/Vpr score, mean	7.72 (± 1.95)
Tat score, mean	2.72(± 1.39)
Vpr score, mean	5.06(± 1.31)
WC, cm	76.1 (± 9.54)
BMI, kg/m^2^ *	21.9 (7.99)
GGT, U/L *	27.5 (52.3)
CRP, mg/L*	2.54 (6.23)
TC, mmol/L	4.05 (± 0.95)
HDL-C, mmol/L	1.12 (± 0.46)
TC: HDL-C, ratio	4.12 (± 1.71)
HbA1c, % *	6.00 (0.50)
Alcohol use (yes), n (%)	23 (38.3)
Tobacco use (yes), n (%)	36 (60.0)
bSBP, mmHg	123 (± 16)
bDBP, mmHg	85 (± 10)
bPP, mmHg	38 (± 10)
bMAP, mmHg	97 (± 12)
HR, bpm	67.0 (± 12.0)
cSBP, mmHg	132 (± 16)
cPP, mmHg	53 (± 11)
cIMT, mm	0.62 (± 0.11)
CSWA, mm^2^	12.6 (± 2.59)
crPWV, m/s	10.9 (± 2.57)

Data are presented as mean (± SD), number of participants (%) or median (IQR) for logarithmically transformed variables, indicated with an asterisk*. Abbreviations: b, brachial; BMI, body mass index; c, central; CD4+, helper T-cells; cIMT, carotid intima media thickness; CRP, C-reactive protein; crPWV, carotid-radial pulse wave velocity; CSWA, cross-sectional wall area; DBP, diastolic blood pressure; GGT, gamma glutamyl transferase; HbA1c, glycated haemoglobin; HDL-C, high density lipoprotein-cholesterol; HR, heart rate; MAP, mean arterial pressure; PP, pulse pressure; SBP, systolic blood pressure; Tat, transactivator of transcription; TC, total cholesterol; Vpr, viral protein R; WC, waist circumference

**Table 2:** Changes observed in the basic characteristics and vascular health measures of PLHIV over a five-year period

	Total population (*N* = 35)			
	Baseline	Follow-up	Difference (baseline – follow-up)	*p*-value
***Metabolic and inflammatory biomarkers***				
BMI, kg/m^2^	24.33 (60.2)	24.36 (7.23)	-0.03 (4.07)	0.096
GGT, U/L	45.03 (53.12)	79.44 (109.75)	-34.41 (92.15)	**0.040**
TC, mmol/L	4.11 (0.93)	4.46 (1.01)	-0.35 (0.81)	**0.017**
HDL-C, mmol/L	1.14 (0.37)	1.43 (0.60)	-0.29 (0.46)	**0.001**
TC: HDL-C, ratio	3.88 (1.27)	3.41 (0.96)	0.47 (0.19)	**0.019**
HbA1c (%)	5.96 (0.40)	5.58 (0.41)	0.38 (0.48)	**< 0.001**
CRP, mg/L	6.86 (12.67)	11.24 (20.23)	-4.38 (21.16)	0.243
***Vascular health measures***				
bSBP, mmHg	121 (17)	122 (26)	-1 (23)	0.082
bDBP, mmHg	84 (11)	81 (14)	3 (16)	**0.028**
bMAP, mmHg	96 (12)	95 (17)	1 (17)	0.554
bPP, mmHg	37 (10)	41 (15)	-4 (13)	0.088
cIMT, mm	0.62 (0.67)	0.59 (0.76)	0.03 (0.08)	**0.017**
CSWA, mm	12.98 (3.51)	12.55 (4.40)	0.43 (3.17)	0.436
Disease status and medication usage*				
Hypertension, (BL +, FU −) / (BL −, FU +)	9 / 7			0.804
cART, (BL +, FU −) / (BL −, FU +)	0 / 27			**< 0.001**

Data are presented as mean (± SD). Values in bold are considered statistically significant (*p* < 0.05). *(BL +, FU −), frequency at baseline positive but negative at follow-up; (BL −, FU +), frequency at baseline negative but positive at follow-up. Abbreviations: b, brachial; BMI, body mass index; cART, combination antiretroviral therapy; cIMT, carotid intima media thickness; CRP, C-reactive protein; CSWA, cross-sectional wall area; DBP, diastolic blood pressure; GGT, gamma glutamyl transferase; HbA1c, glycated haemoglobin; HDL-C, high density lipoprotein-cholesterol; MAP, mean arterial pressure; PP, pulse pressure; SBP, systolic blood pressure; TC, total cholesterol

### Cross-sectional associations of Tat and Vpr sequence variation with vascular health measures

**Fig. 2:**
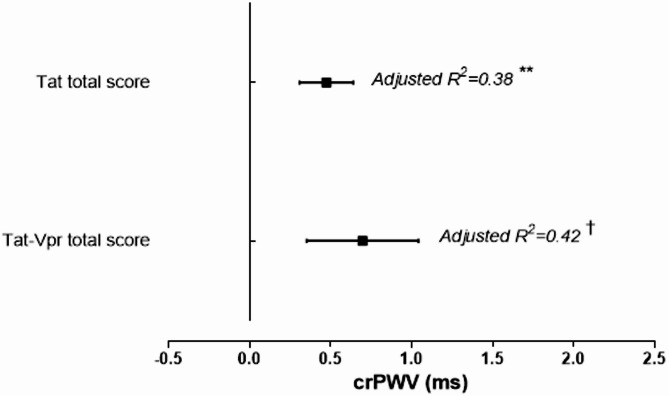
Independent associations of baseline crPWV with the Tat only and Tat/Vpr scores after adjusting for age, sex, locality, CRP and bMAP. Superscript symbol shows the trend of significance: *p ≤ 0.05, **p ≤ 0.01, †*p* ≤ 0.001. Abbreviations: crPWV, carotid-radialis pulse wave velocity; Tat, Trans-activator of transcription; Vpr, Viral protein R

Backward stepwise regression analyses were performed to explore associations independent of age, sex, locality, CRP and bMAP (in the case of crPWV and cIMT only). No significant associations were found for the Vpr only score. In the total cohort (Fig. 2), significant associations were observed between the Tat only score (adj R^2^ = 0.38, β = 0.47, *p* = 0.002) and the Tat/Vpr score (adj R^2^ = 0.42, β = 0.70, *p* < 0.001) with crPWV. No significant associations were found for any of the other vascular health measures (bSBP, bDBP, bPP, bMAP, cIMT, CSWA, cSBP, cPP) in the total cohort. Variation in the baseline Tat and Vpr protein sequence did not increase probability for hypertension at baseline (all models *p* > 0.27).

### Longitudinal associations of Tat and Vpr sequence variation with vascular health measures and five-year all-cause mortality risk

Backward stepwise regression analyses were conducted to examine the associations within the total cohort with available follow-up data (*N* = 35) independent of locality, BMI, GGT, cART medication use and bMAP (in the case of cIMT only). No significant associations were found for any of the vascular health measures (bSBP, bDBP, bPP, bMAP, cIMT, CSWA). Variation in the baseline Tat and Vpr protein sequence did not increase the risk for incident hypertension, nor did it predict the risk of all-cause mortality after five years in this study (all models *p* > 0.15).

## Discussion

The aim of this study was to investigate the relationship between the amino acid sequence variation of Tat and Vpr viral proteins with vascular health measures and to determine whether amino acid sequence variation predicts five-year incident hypertension and all-cause mortality in a cohort of South African PLHIV. This study found that Tat and Vpr sequence variations were linked to baseline crPWV but not to longitudinal changes, five-year incident hypertension, or all-cause mortality in PLHIV.

We showed that Tat/Vpr and Tat only amino acid sequence variations associate with crPWV cross-sectionally. To our knowledge, no previous studies have reported direct associations between arterial stiffness and Tat or Vpr with studies only identifying potential indirect connections. The expression of HIV proteins, such as Tat and Vpr, coincided with the presence of pre-clinical markers of atherosclerosis and correlated with alterations in markers of vascular remodelling, irrespective of highly active ART or HIV infection in mice [35]. This is supported by the observation that crPWV showed no significant difference between HIV-positive, ART-naïve participants, and age- and sex-matched HIV-negative participants (see Supplementary Table 2), nor between HIV-positive, ART-naïve participants and those receiving treatment [36]. Vpr was shown to suppress the activity of peroxisome proliferator-activated receptor-gamma (PPARγ) [37], which could lead to vascular remodelling and endothelial dysfunction through enhanced vascular oxidative stress and inflammation [38]. Further research conducted by our team has demonstrated that amino acid variations in Vpr, specifically at position 41 (between Glycine 41 and Serine 41) and 55 (between Alanine 55 and Threonine 55), are linked to soluble urokinase plasminogen activator receptor (suPAR) [31] and quinolinic acid, key markers implicated in CVD [39, 40].

Furthermore, Tat could enhance endothelial dysfunction through endothelial cell expression of various adhesion molecules, including soluble intracellular adhesion molecule (ICAM-1) and soluble vascular cell adhesion molecule (VCAM-1) [41, 42], possibly by potentiating the TNF-mediated activation of nuclear factor kappa-light-chain-enhancer of activated B cells (NF-κB) [42]. Although the underlying mechanism of these associations was beyond the scope of our study, previous findings in our cohort showed that adhesion molecules do not associate with cIMT and crPWV over a period of five years, even though these adhesion molecules were elevated in PLHIV at baseline [43]. Unexpectedly, cIMT decreased in this study, consistent with a previous observation in the same cohort [44]. This decline was suggested to be attributed to the use of ART and a more favourable lipid profile, as cIMT at follow-up (2015) was significantly associated with baseline TG: HDL-C ratio and inversely with ART use [43]. The observed decrease in cIMT in this study may be attributed to the decrease in the TC: HDL-C ratio and cART initiation [45]. However, as we found no associations with viral proteins, further research is needed to provide a definitive explanation for the improvement. Furthermore, no significant associations were found between Tat amino acid residues and markers of endothelial activation and inflammation in a separate cohort of South African individuals of African descent [46]. Therefore, the mechanism that aims to explain the cross-sectional association between Tat and arterial stiffness may not involve Tat-induced endothelial dysfunction and requires further investigation in PLHIV of African descent. Additionally, since PWV did not differ between HIV-positive, ART-naïve participants and age- and sex-matched HIV-negative participants (Supplementary Table 2) or those on treatment [36], it remains unclear whether arterial stiffening differs between these groups over time or if viral proteins contribute to this process.

Lastly, we investigated whether Vpr and Tat amino acid sequence variation predicted incident hypertension and all-cause mortality over a five-year period. Since a limited number of participants developed incident hypertension (*N* = 7) or succumbed during this timeframe (*N* = 9), this could have prevented robust predictive conclusions. In particular, no participants experienced mortality solely attributable to cardiovascular reasons. Most studies on the impact of HIV-1 subtype variation and amino acid sequence diversity on CVD are underexplored, with existing evidence mainly from regions where subtype B predominates. The results if this study underscore the need for larger cohort studies to better understand the relationships between viral protein variations and mortality, as research is currently limited.

### Strengths and limitations

The main limitation of our study was the small sample size, which may have been underpowered to detect specific associations. Although 125 participants were initially enrolled, only 60 yielded usable viral sequence data, and 35 completed follow-up. This reduction was primarily due to sequencing failure, which stemmed from technical constraints associated with the use of archived plasma samples. RNA was extracted and reverse transcribed for Sanger sequencing; however, this method is less effective when RNA integrity is compromised, as is often the case with stored specimens. Furthermore, the high genetic diversity of HIV presents additional challenges for reliable amplification and sequencing. The use of cellular material, such as buffy coat, could have improved sequencing yield by providing access to higher-quality nucleic acids, but was not possible in this study due to the retrospective nature of sample collection. There was a relatively high loss to follow-up within the sub-sample of participants who had successful viral sequencing (27% excluding deaths). This level of attrition introduces a potential risk of selection bias, as individuals who completed follow-up may differ from those who were lost, thereby affecting the generalizability of the findings. Unfortunately, such attrition is a well-documented challenge in longitudinal studies in African settings, often driven by high rates of residential mobility and migration [47, 48]. Consequently, both the small final sample size and the possibility of selection bias due to attrition should be carefully considered when interpreting the results. Our investigation focused only on two key proteins, Tat and Vpr, while sequence variations in other viral proteins, such as gp120, may also impact clinical outcomes in PLHIV [49]. The effect of sequence variations in these other proteins on vascular health measures remains unclear. Viral protein sequencing was only successful for 50% of participants who were not on cART (Fig. 1), and baseline differences between participants with successful sequencing and those without (Supplementary Table 1), specifically in cIMT and cPP, could have influenced our findings. The use of different devices and arterial segments to measure stiffness in the 2010 and 2015 phases limited our ability to calculate changes in PWV, cSBP, and cPP. This hindered our ability to evaluate the longitudinal relationship of the significant findings observed for PWV in the cross-sectional analysis. There is conflicting evidence regarding the role of viral load (viremia), specifically in relation to arterial stiffness [50]. The PURE study did not assess HIV viral load, so its potential impact on arterial stiffness could not be evaluated. We also had data on cART initiation dates for only 37% of participants, limiting our ability to assess treatment duration effects. At the time of data collection, some older cART regimens, such as stavudine, which is no longer in use, were still being prescribed. The outdated cART regimens may have contributed to cardiovascular risk through metabolic side effects, including dyslipidemia, lipodystrophy, and the development of metabolic syndrome. Although we did not directly assess lipodystrophy, our analyses accounted for several components of metabolic dysfunction by including markers such as TC: HDL-C ratio, body mass index (BMI), HbA1c, and blood pressure (when not the primary outcome). Among these, only BMI entered the final models in longitudinal analyses, while lipid measures and HbA1c did not significantly contribute to either cross-sectional or longitudinal models. These findings suggest that while metabolic changes related to historical ART exposure are an important consideration, they were unlikely to fully account for the observed associations in this study. Nonetheless, this remains a limitation and should be interpreted within the context of evolving cART regimens and their known systemic effects. Therefore, future studies should aim to validate these findings in larger cohorts receiving current first-line cART treatments including nucleoside reverse transcriptase inhibitors (NRTI) and non-nucleoside reverse transcriptase inhibitors (NNRTI). The lack of family history data and the unknown duration since HIV infection are further limitations.

While the 2025 UNAIDS treatment targets may question the relevance of studying an ART-naïve population, South Africa’s progress toward 90% ART coverage is still limited, particularly in rural areas where 50% of our cohort resides. This highlights the need to study viral proteins’ role in CVD in underserved regions. Our sample, consisting of ART-naïve individuals at the time of data collection, offers unique insights into HIV proteins’ relationship with arterial stiffness without cART. To our knowledge, this is the first study to report on Tat and Vpr amino acid variation and arterial stiffness in treatment-naïve PLHIV.

## Conclusion

In this exploratory study, we examined potential associations between Tat and Vpr sequence variants and vascular health measures. Our findings suggest that sequence variation in Tat and Vpr may be associated with pulse wave velocity, a marker of arterial stiffness. These results are intriguing but should be interpreted cautiously due to the small sample size. Further research is needed to confirm these associations and explore the role of HIV-1 protein variants in CVD development.

## Supplementary Information

Below is the link to the electronic supplementary material.

Supplementary Material 1

## Data Availability

The datasets generated and/or analysed during the current study are available from the corresponding author on reasonable request. All Tat and Vpr sequences can be accessed in GenBank under the accession numbers OR621303-OR621349 for Vpr and OR645413-OR645449 for Tat.
