# Supplementary material for: Variation in HIV-1 Tat and Vpr protein amino acid sequences and its association with vascular health measures in a South African cohort: an exploratory study
Source: Virol J. 2025 Aug 4;22:266. doi: 10.1186/s12985-025-02891-8 (PMC12323018; doi:10.1186/s12985-025-02891-8)
Supplement: Supplementary file 1 — Supplementary Material 1 [file 12985_2025_2891_MOESM1_ESM.docx]

**Supplementary Table 1:** Baseline Demographic and Cardiometabolic Characteristics of HIV-Positive Participants by HIV-1 Viral Protein Sequencing Status

|  | **Successfully sequenced HIV-1 viral proteins**  **(N=60)** | **Could not be sequenced**  **(N=65)** | **p-value** |
| --- | --- | --- | --- |
| **Sex (male), n (%)** | 16 (26.7) | 20 (30.3) | 0.613 |
| **Locality (rural), n (%)** | 31 (51.7) | 31 (47.0) | 0.657 |
| **Age, mean *** | 46.00 (8.00) | 48.00 (10.00) | **0.049** |
| **CD4+ Nadir, cells/mm^3^** | 302 (±149) | 368 (±226) | 0.158 |
| **BMI, kg/m^2^ *** | 21.80 (8.00) | 22.30 (8.00) | 0.905 |
| **GGT, U/L *** | 27.50 (52.28) | 38.00 (59.40) | 0.893 |
| **CRP, mg/L*** | 2.54 (6.23) | 3.28 (10.49) | 0.285 |
| **TC, mmol/L** | 4.05 (±0.95) | 4.49 (±1.13) | **0.023** |
| **HDL, mmol/L** | 1.12 (±0.46) | 1.21 (±0.41) | 0.251 |
| **Glucose, mmol/L *** | 4.62 (0.69) | 5.15 (0.78) | 0.635 |
| **HbA1c, % *** | 6.00 (1.00) | 5.80 (1.00) | 0.151 |
| **Alcohol use (yes), n (%)** | 23 (38.3) | 24 (36.4) | 0.270 |
| **Tobacco use (yes), n (%)** | 36 (60.0) | 29 (43.9) | 0.185 |
| **bSBP, mmHg** | 123 (±16) | 124 (±21) | 0.597 |
| **bDBP, mmHg** | 85 (±10) | 84 (±13) | 0.903 |
| **bPP, mmHg** | 38 (±10) | 40 (±13) | 0.324 |
| **bMAP, mmHg** | 97 (±12) | 98 (±15) | 0.847 |
| **HR, bpm** | 67 (±12.0) | 69 (±16) | 0.478 |
| **cSBP, mmHg** | 132 (±16) | 137 (±22) | 0.190 |
| **cPP, mmHg** | 53 (±11) | 58 (±14) | **0.023** |
| **cIMT, mm** | 0.61 (±0.09) | 0.67 (±0.17) | **0.042** |
| **CSWA, mm^2^** | 12.58 (±2.59) | 14.12 (±5.57) | 0.056 |
| **crPWV, m/s** | 10.89 (±2.57) | 10.40 (±2.15) | 0.265 |
| **HT status, n (%)** | 26 (41.3) | 24 (40.0) | 0.886 |
| **Use of antihypertensive medication (yes), n (%)** | 4 (6.7) | 15 (23.1) | **0.011** |
| **Use of diabetic medication (Yes), n (%)** | 1 (1.7) | 1 (1.5) | 0.954 |

Data are presented as mean (±SD), number of participants (%) or median (IQR) for logarithmically transformed variables, indicated with an asterisk*. Values in bold are considered statistically significant (p<0.05).

**Supplementary Table 2:** Baseline Demographic and Cardiometabolic Characteristics of HIV-Positive Participants for which HIV-1 Viral Protein Sequencing was done compared to aged and sex-matched HIV-negative Participants

|  | **HIV-positive participants**  **(N=60)** | **Age and sex-matched HIV-negative participants**  **(N=59)** | **p-value** |
| --- | --- | --- | --- |
| **Sex (male), n (%)** | 16 (26.7) | 15 (25.4) | 0.877 |
| **Locality (rural), n (%)** | 31 (51.7) | 5 (8.5) | **<0.001** |
| **Age, mean *** | 46.00 (8.00) | 46.00 (8.00) | 0.909 |
| **BMI, kg/m^2^ *** | 21.80 (8.00) | 28.70 (15.00) | **<0.001** |
| **GGT, U/L *** | 27.50 (52.28) | 35.00 (61.90) | 0.403 |
| **CRP, mg/L*** | 2.54 (6.23) | 4.07 (5.71) | 0.569 |
| **TC, mmol/L** | 4.05 (±0.95) | 4.82(±1.18) | **<0.001** |
| **HDL, mmol/L** | 1.12 (±0.46) | 1.44 (±0.49) | **<0.001** |
| **Glucose, mmol/L *** | 4.62 (0.69) | 5.17 (0.90) | **0.011** |
| **HbA1c, % *** | 6.00 (1.00) | 6.00 (1.00) | 0.450 |
| **Alcohol use (yes), n (%)** | 23 (38.3) | 23 (39.0) | 0.270 |
| **Tobacco use (yes), n (%)** | 36 (60.0) | 32 (52.5) | 0.705 |
| **bSBP, mmHg** | 123 (±16) | 130 (±22) | **0.030** |
| **bDBP, mmHg** | 85 (±10) | 88 (±12) | 0.098 |
| **bPP, mmHg** | 38 (±10) | 42 (±14) | 0.065 |
| **bMAP, mmHg** | 97 (±12) | 102 (±15) | **0.043** |
| **HR, bpm** | 67 (±12) | 67 (±13) | 0.867 |
| **cSBP, mmHg** | 132 (±16) | 140 (±24) | **0.042** |
| **cPP, mmHg** | 53 (±11) | 58 (±16) | **0.030** |
| **cIMT, mm** | 0.61 (±0.09) | 0.66 (±0.11) | **0.007** |
| **CSWA, mm^2^** | 12.58 (±2.59) | 13.93 ± (3.26) | **0.016** |
| **crPWV, m/s** | 10.89 (±2.57) | 10.63 (±2.45) | 0.593 |
| **HT status, n (%)** | 26 (41.3) | 24 (40.0) | 0.886 |
| **Use of antihypertensive medication (yes), n (%)** | 4 (6.7) | 11 (19.0) | **0.045** |
| **Use of diabetic medication (Yes), n (%)** | 1 (1.7) | 1 (1.8) | 0.971 |

Data are presented as mean (±SD), number of participants (%) or median (IQR) for logarithmically transformed variables, indicated with an asterisk*. Values in bold are considered statistically significant (p<0.05).
